# Development and Optimization of Instant Cassava‐Soybean Composite Porridge as a Nutrient‐Enhanced Complementary Food for Children Aged 6–59 Months

**DOI:** 10.1002/fsn3.71957

**Published:** 2026-05-28

**Authors:** Robert Mugabi, Catherine Byekwaso Arinaitwe, Ivan Muzira Mukisa

**Affiliations:** ^1^ Department of Food Technology and Nutrition, School of Food Technology, Nutrition and Bioengineering Makerere University Kampala Uganda

**Keywords:** cassava‐soybean composite, complementary food, extrusion cooking, infant nutrition, optimization, protein digestibility

## Abstract

Childhood malnutrition remains a significant challenge in sub‐Saharan Africa, particularly in regions where cassava is a staple. Despite its prevalence, cassava is an inadequate complementary food for children aged 6–59 months due to its low protein content, persistent cyanogenic glucosides, and high‐viscosity, low‐nutrient density. This study aimed to develop and optimize an instant cassava‐soybean composite porridge using extrusion cooking. Using a mixture design to evaluate nine formulations against WHO nutrient recommendations, a 70:30 cassava‐to‐soybean ratio was identified as optimal. A central composite design involving 21 experimental runs further optimized extrusion conditions by varying barrel temperature (BT) (60°C–150°C) and feed moisture (16%–30%). The ideal extrusion parameters were determined to be a 145.6°C (BT) and 19.7% feed moisture. The resulting flour contained 12.04% protein with 70.9% digestibility, 8.42% moisture, and a viscosity of 1086.63 cP. Notably, extrusion significantly reduced cyanogenic glucosides, enhancing product safety. Sensory evaluation by a semi‐trained panel of 30 adults using a 9‐point hedonic scale indicated high consumer appeal, with overall acceptability and texture scores of 7.3 and 7.4, respectively (𝑝 < 0.05). Cost analysis showed the final product is 56%–65% cheaper than commercial alternatives like Cerelac. In summary, this optimized porridge complies with WHO guidelines for complementary foods, offering a feasible, safe, and economical nutritional solution for cassava‐consuming communities.

## Graphical abstract

This study details the development and optimization of an affordable, nutrient‐dense instant complementary porridge formulated from a 70:30 composite blend of locally sourced cassava (NaroCas2) and soybean (Maksoy1N). Utilizing Response Surface Methodology (RSM), optimal twin‐screw extrusion parameters were established at a barrel temperature of 145.6°C and 19.7% feed moisture content. Under these optimized conditions, the resulting shelf‐stable flour yielded 12.04% crude protein with an improved in vitro protein digestibility of 70.9%. Furthermore, starch modifications effectively reduced porridge viscosity to 1086.63 cP, well within the WHO threshold (< 2500 cP) to ensure high energy density and ease of swallowing for young children. This optimized local formulation demonstrates high sensory acceptability and offers a 56%–65% cost advantage over imported commercial alternatives like Cerelac.

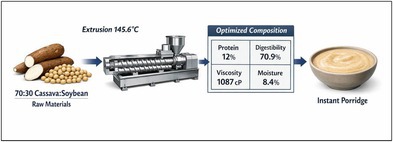

## 1 Introduction

Childhood malnutrition remains a serious public health problem globally. Approximately 148.1 million children under 5 years are stunted and 45 million are wasted (UNICEF/WHO/World Bank 2023). In sub‐Saharan Africa, poor complementary feeding practices are a major cause of undernutrition, and in Uganda, approximately 29% of children aged 6–59 months are stunted (Uganda Bureau of Statistics and ICF 2022), because the critical window of complementary feeding (6–24 months) is the most important period for determining growth and development, and any growth faltering and developmental delays during this period are often irreversible (UNICEF 2024; WHO 2023). Cassava ( *Manihot esculenta* Crantz) is the third most important staple crop in Uganda in terms of its contribution to food security, after maize and bananas (FAOSTAT 2019; Marenya et al. 2012), and it is preferred because of its ability to withstand drought and grow well in poor soils (Montagnac et al. 2009), making it an important crop for subsistence farmers. However, cassava roots are composed of 80%–90% water, and the dry matter is mainly made up of carbohydrates (80%–90%) and small amounts of protein (1%–2%) and essential micronutrients (Montagnac et al. 2009), thus cassava alone cannot meet the nutritional needs of infants, which require 13 g protein and 902–1046 kcal energy per day (WHO 2012). Soybean ( *Glycine max* ) is a legume rich in protein (40%–42%) with a good amino acid profile, particularly lysine, which is limiting in cereals and root crops (Sharma et al. 2014; Banaszkiewicz 2011), therefore, combining soybean with cassava can enhance the protein quality of the diet through amino acid complementation and address protein‐energy malnutrition in cassava‐dependent communities (Abbas and Ahmad 2018). Recent evidence shows that 181 million children under five globally live in severe child food poverty, with Sub‐Saharan Africa disproportionately affected (UNICEF 2024), and a 10‐year review of complementary food development in sub‐Saharan Africa highlights the urgent need for locally produced, nutrient‐dense foods (Ogunniran et al. 2024).

Extrusion cooking is a promising technology for the production of complementary foods, because it improves nutrient bioavailability, reduces the levels of anti‐nutritional factors, enhances digestibility, extends shelf life, and produces instant, ready‐to‐eat products with low bulk density and appropriate viscosity for infants (Oladiran and Emmambux 2022; Barrows et al. 2007), while the HTST conditions during extrusion induce starch gelatinization and protein denaturation, which improve digestibility and viscosity (suitable for infant feeding) (Licata et al. 2014). However, the extrusion conditions must be carefully controlled, because excessive heat and unsuitable moisture can increase Maillard reactions, reducing the available lysine content and ultimately the protein quality (Barrows et al. 2007), and despite the potential of cassava‐soybean composite flours, there is a lack of systematic work to optimize the extrusion conditions for better nutritional quality, while maintaining good sensory properties and appropriate physical characteristics, since most of the previous work has focused on cereal‐legume combinations and less on root‐legume combinations, with little or no attention paid to cassava‐based formulations. The majority of complementary foods on the Ugandan market are imported and are not affordable to low‐income households, thus there is a need for development of nutritious, locally produced, and affordable products, and the objective of this study was to fill this knowledge gap by optimizing both the cassava‐soybean composite flour and the extrusion conditions using response surface methodology. The objectives of this study were to: (1) develop cassava‐soybean composite flours that meet the nutritional requirements of children aged 6–59 months; (2) determine the optimal extrusion conditions (Barrel temperature [BT] and feed moisture content [FMC]) that maximize protein content, protein digestibility (PD), and sensory acceptability, while maintaining suitable viscosity; and (3) characterize the nutritional profile and physicochemical properties of the optimized product and compare it with existing commercial complementary foods. Recent studies have demonstrated the potential of extrusion technology for developing affordable complementary foods from locally available ingredients in sub‐Saharan Africa (Oladiran and Emmambux 2022; Ogunniran et al. 2024; Masuke et al. 2021), and the WHO (2023) updated guidelines on complementary feeding emphasize the importance of nutrient‐dense, locally produced foods for children aged 6–23 months.

## 2 Materials and Methods

### 2.1 Raw Materials and Chemicals

Fresh cassava roots (NaroCas2 variety), a sweet and low cyanide variety, were obtained from National Crops Resources Research Institute (NaCRRI) Namulonge, Uganda, and soybean grains (Maksoy1N variety) were obtained from Makerere University Agricultural Research Institute, Kabanyolo (MUARIK), Kampala. Analytical grade reagents and chemicals were purchased from Neo Faraday Laboratory Supply in Kampala, and the cassava roots, soybeans, and other materials were transported to the laboratory at School of Food Technology, Nutrition and Bioengineering, Makerere University, where they were stored under appropriate conditions until used for processing.

### 2.2 Experimental Design and Formulation Optimization

#### 2.2.1 Formulation Development

A nutrient‐balanced composition of the cassava‐soybean composite was established through the Scheffé simplex lattice mixture design in Design Expert (Version 11, Stat‐Ease Inc., Minneapolis, USA) and NutriSurvey (Erhardt 2007). Based on the WHO recommendation for children aged 6–59 months (WHO 2012), the target specifications were determined, requiring at least 13 g of protein per day and an energy intake range of 902–1046 kcal/day. As suggested by Cornell (2002), a mixture design was applied because it was the best choice for optimization problems whose response depends upon the ratio of constituents instead of their quantity.

For the present study, the boundaries for the ratio of cassava and soybean were defined from 86:14 to 70:30. The highest ratio of 86% was limited by sensory properties (less than 5.0 on the 9‐point scale) and economic constraints, as the composite flours exceeding the value resulted in less than 10 g/100 g of protein required to meet the daily protein intake (WHO 2012). On the contrary, the 70:30 ratio was chosen as the lower boundary to provide a safety margin. Though formulations like 75:25 had the borderline amount of protein, the latter was essential for accounting for the possible extrusion losses from 5% to 15% (Alam et al. 2016).

Among all nine formulations, only three (2, 6, and 9) were found to satisfy the nutritional requirements. All formulations were evaluated based on NutriSurvey analysis and USDA food composition data for nutrients predictions. Finally, formulation 9, consisting of 70% cassava flour and 30% soybean flour, was selected as the best one, providing the maximum protein amount (14.0 g/100 g), meeting the energy requirements (306.6 kcal/100 g), and showing good sensory acceptance scores. Thus, the 70:30 ratio was recommended for optimization in the extrusion process (see Table 1).

**TABLE 1:** Cassava and soybean flour ratios evaluated to determine the optimal combination (*n* = 3 replicate analyses per formulation).

Formulation	Cassava (%)	Soy (%)	Energy (kcal)	Protein (g)	Iron (mg)
1	86.4	13.6	328.7	6.6	2.5
2	86.0	14.0	328.0	6.8	2.5
3	80.0	20.0	312.9	9.5	3.2
4	79.0	21.0	317.4	10.0	3.3
5	78.5	21.5	316.6	10.2	3.4
6	78.0	22.0	315.8	10.4	3.4
7	76.0	24.0	312.8	11.4	3.6
8	72.0	28.0	306.7	13.1	4.1
9	70.0	30.0	306.6	14.0	4.3

#### 2.2.2 Extrusion Process Optimization

A central composite (I‐optimal) design with two factors (BT and FMC) was used to optimize the extrusion conditions, and the ranges of the factors were determined from the preliminary trials as follows: BT (60°C–150°C) and FMC (16%–30%). Below 60°C, the product was not well cooked, while above 150°C, the product was charred (Oladiran and Emmambux 2022). Similarly, below 16% moisture content, the product was too dry and hard, while above 30% moisture content, the stability of the product was negatively affected (Makowska et al. 2018).

Twenty‐one experiments were performed with four experiments at the center point to enable the estimation of pure error, and the responses were protein content (PC), protein digestibility (PD), moisture content (MC), and viscosity. The data were fitted to a second‐order polynomial model (Equation 1) to describe and predict the effect of BT and FMC on the product characteristics.

(1) $$Y=\beta_{o}+\sum_{i-1}^{k}\beta_{i}X_{i}+\sum_{i-1}^{k}\beta_{\mathrm{ii}}X_{i}^{2}+\sum_{\begin{array}{c} i-1\\ i<j \end{array}}^{k}\beta_{\mathrm{ij}}X_{i}X_{j}+\varepsilon$$

where *Y* represents the response variable, *X_i_* and *X_j_* are independent variables (BT and FMC), *β*₀ is the intercept, *β_i_*, *β_ii_*, and *β_ij_* are regression coefficients for linear, quadratic, and interaction effects, respectively, and *ε* denotes random error.

### 2.3 Sample Preparation

#### 2.3.1 Preprocessing of Raw Materials

Soybean grains were hand sorted to remove stones, debris, and damaged seeds and then roasted at 140°C for 20 min in an infrared food oven (Model GU‐6, New Zealand) because the roasting of soybeans was done to reduce the level of anti‐nutritional factors, especially trypsin inhibitors and lipoxygenase (Abbas and Ahmad 2018).

Fresh cassava tubers were washed under running tap water, manually peeled to remove the outer cortex, sliced into chips of about 2 mm thickness using a manual slicer, and sun dried for 72 h until the moisture content was reduced to about 10%–12%. The roasted soybean and dried cassava chips were separately milled into fine flour (particle size < 0.5 mm) using a locally fabricated hammer mill.

#### 2.3.2 Extrusion Cooking

Extrusion cooking involved mixing cassava and soybean flours in a 70:30 ratio, and then the moisture content of the composite flours was adjusted to the required levels (16%–30%) by the addition of water to the composite flour under continuous mixing. The composite floured and moistened samples were then sealed in polyethylene bags and allowed to equilibrate for 2 h in order to allow the water to distribute uniformly in the flour, and then extrusion was carried out in a co‐rotating twin‐screw extruder (Quitong‐Kneader Model JS‐60D, China) of 45 mm screw diameter, 550 mm length, 3:1 compression ratio and 3 mm die diameter. The operating conditions were kept constant for all the runs, including a screw speed of 30.9 rpm, feed rate of 36.1 kg/h, cutter speed of 22.8 rpm, and residence time of about 2 min, because the barrel consisted of three heating zones, and the temperature in the three zones was adjusted according to the experimental design (Table 2). The twin‐screw extrusion approach was selected based on its demonstrated effectiveness in producing uniform, high‐quality extrudates from cereal‐legume composite flours (Alam et al. 2016; Gbenyi et al. 2023; Masatcioglu et al. 2022).

**TABLE 2:** Processing variables of corn‐soy and their levels.

Variables	Symbol	Coded variables		
		−1	0	+1
Feed moisture (%)	*X*_1_	16	25	30
BT (°C)	*X*_2_	60	100	150

Extrudates were collected immediately after exiting from the die and dried in an oven (50°C for 30 min) to improve crispiness and sensory attributes, and they were then allowed to cool to room temperature (25°C ± 2°C) for about 10 min. Cooled extrudates were milled (Model 30B‐C) using a 0.6 mm mesh to produce instant flour, which was then packed in sealed polyethylene bags and stored at 4°C until analysis.

### 2.4 Analytical Methods

#### 2.4.1 Proximate Composition

The moisture content of the sample was determined by drying the sample in a hot air oven at 105°C to constant weight (AOAC 1999; Method 925.10), and crude protein content was determined by the Kjeldahl method (AOAC 1990; Method 960.52). Nitrogen content was determined by Kjeldahl digestion of the sample (420°C for 1 h) in the presence of a catalyst mixture of K_2_SO_4_:CuSO_4_ (10:1) with concentrated sulfuric acid, followed by distillation of the ammonia into boric acid and titration with standardized hydrochloric acid; therefore the nitrogen content was calculated as follows: *N* (%) = ((*V*_sample_ − *V*_blank_) × *N*_acid_ × 14.007)/(*W* × 10); where *V*_sample_ represents the volume (mL) of standardized HCl used in titrating the sample, *V*_blank_ is the volume (mL) used for the blank, *N*_acid_ is the normality of the HCl solution (mol/L), 14.007 is the atomic weight of nitrogen (g/mol), and *W* is the sample weight (g). The crude protein content was then estimated by multiplying the nitrogen content by a factor of 6.25: Protein (%) = *N* (%) × 6.25; this conversion factor is commonly applied to mixed plant‐based foods, following standard AOAC recommendations (1990). The total fat content of the sample was determined by Soxhlet extraction using petroleum ether as solvent (AOAC 1999; Method 920.39), while ash content of the sample was determined by incineration in a muffle furnace at 550°C for 6 h (AOAC 1990; Method 923.03). Total dietary fiber content was determined using an enzymatic‐gravimetric method with the FOSS Fibertec 2010 system, and total carbohydrate content was then determined by difference: Carbohydrate (%) = 100 − (% moisture + % protein + % fat + % ash + % fiber). All proximate analyses were performed in triplicate (*n* = 3).

#### 2.4.2 Energy Content

Gross energy of the samples was determined using an oxygen bomb calorimeter (Gallenkamp Autobomb) as described by AOAC (1999) Method 945.39, and metabolizable energy was estimated using the Atwater factors as in Equation 2.

(2) $$\text{Energy}\;\left(\text{kcal}/100g\right)=\left(\text{Protein}\times 4\right)+\left(\text{Carbohydrate}\times 4\right)+\left(\mathrm{Fat}\times 9\right)$$

#### 2.4.3 In Vitro PD

PD was estimated by employing a two‐step enzymatic digestion method, as described by Chavan et al. (2001) and Saunders et al. (1973), which involved digesting protein with pepsin (pH 2.0, 37°C for 3 h) followed by pancreatin (pH 7.5, 37°C for 24 h), and after the digestion process, the nitrogen content in the supernatant was estimated and the percentage of digestible protein was calculated. All PD measurements were performed in triplicate (*n* = 3).

#### 2.4.4 Physicochemical Properties

The swelling power and solubility of the flour samples were determined according to the modified method described by Li and Yeh (2001), and briefly, a 1% (w/v) flour suspension was heated at 85°C for 30 min with constant stirring, and then centrifuged at 3000 rpm for 15 min. The swelling power was calculated based on the weight of swollen sediment, while the solubility was calculated based on the solids content of the dried supernatant. The viscosity of the reconstituted porridge (20% w/v flour in water) was measured at 50°C using a Brookfield viscometer (Model DV‐II + Pro) equipped with spindle number 4 at 50 rpm, and the pasting properties of the flours were evaluated using a Rapid Visco Analyzer (RVA‐4, Newport Scientific, Australia) according to AACC Method 76–21. All physicochemical measurements were performed in triplicate (*n* = 3), and results are reported as mean ± standard deviation (SD).

#### 2.4.5 Sensory Evaluation

The sensory evaluation was conducted using 30 semi‐trained panelists (15 males and 15 females, 20–45 years) selected from the Department of Food Technology and Nutrition, Makerere University, and panelists were screened for any known allergies to soy and cassava products. The panelists were also trained on the use of a 9‐point hedonic scale (1 = dislike extremely, 9 = like extremely) before the actual test, and then the samples were prepared by mixing 50 g of each extruded flour with 200 mL of boiling water. The composite flour was stirred continuously until a smooth consistency was attained, and then the porridge was allowed to cool to about 45°C before serving. Approximately 30 mL of each sample was served in coded white plastic cups under normal fluorescent lighting at room temperature (25°C ± 2°C), and the panelists evaluated the samples for appearance, aroma, taste, texture/mouthfeel, and overall acceptability. The order of serving was randomized to avoid bias, and water was provided to panelists between samples to cleanse the palate; therefore, all sensory evaluations were done in triplicate (*n* = 3). Sensory data were analyzed using one‐way analysis of variance (ANOVA) followed by Tukey's honestly significant difference (HSD) post hoc test (*p* < 0.05) to determine significant differences among sensory attributes across formulations, using IBM SPSS Statistics (Version 26, IBM Corp., Armonk, NY, USA). Evaluations were conducted in individual sensory booths to minimize interaction among panelists and reduce bias. Ethical approval for the sensory evaluation was obtained from the Makerere University, School of Food Technology, Nutrition and Bioengineering—Research and Higher Degrees Committee (Reference No. SFTNB‐2023‐045). All panelists provided written informed consent prior to participation.

### 2.5 Optimization and Statistical Analysis

All responses were optimized simultaneously using the desirability function approach (DFA), with a desirability function defined for each response, setting PD and sensory acceptability to the maximum, viscosity to the minimum acceptable value for infant feeding (< 2500 cP), and protein content to the maximum. The overall desirability (D) was then obtained as the geometric mean of all the individual desirability values, and statistical analysis was done using Design Expert software (Version 11), because analysis of variance (ANOVA) was used to check if the models were significant, with *p* < 0.05 used as the cut‐off for statistical significance. Moreover, model performance and adequacy were checked using *R* ^2^, adequate precision, and the lack‐of‐fit test, and therefore, response surface and contour plots were generated to illustrate the effects of BT and feed moisture on each response.

## 3 Results and Discussion

### 3.1 Effect of Extrusion Parameters on Product Characteristics

#### 3.1.1 Moisture Content

The moisture content of the extruded cassava‐soybean samples varied from 7.8% ± 0.3% to 19.1% ± 0.5% (Table 3). The lowest moisture content was observed for the product extruded at high BT and low feed moisture (150°C, 16% FMC), and the highest moisture content was observed at low BT and high feed moisture (60°C, 30% FMC); therefore, the moisture content was well predicted by the second‐order polynomial model (*R* ^2^ = 0.955; Equation 3), which indicated that the model explained most of the variation in moisture content.

(3) $$\mathrm{MC}=12.46-5.03X_{1}+1.70X_{2}+1.42X_{1}X_{2}+0.59X_{1}2+2.89X_{2}2$$

where *X*_1_ represents FMC and *X*_2_ represents BT. All model terms were statistically significant (*p* < 0.05), with negligible lack of fit (*p* > 0.05), indicating excellent model validity.

**TABLE 3:** Experimental design of FMC and BT (*n* = 3 replicates per experimental run; values expressed as mean ± SD).

Run	Barrel temperature (°C)	Feed MC (%)	MC (%)	PC (%)	PD (%)	Viscosity (cP)
1	118.95	30.0	14.8	12.6	66.9	1990
2	150.0	30.0	12.3	12.7	70.6	1099
3	105.0	23.0	11.4	13.5	69.2	1690
4	60.0	16.0	18.0	12.9	58.3	2016
5	119.4	16.0	13.0	14.1	64.7	1489
6	150.0	16.0	8.3	10.6	69.8	1044
7	82.5	20.2	16.1	12.7	65.9	2000
8	150.0	23.0	8.7	11.9	69.9	1051
9	60.0	23.0	17.1	12.9	58.8	2094
10	105.0	23.0	11.6	13.8	69.2	1694
11	150.0	23.0	8.9	12.0	69.6	1049
12	90.4	16.0	14.8	12.2	59.9	2010
13	105.0	23.0	11.0	13.6	69.1	1699
14	60.0	30.0	19.0	12.7	60.1	2106
15	150.0	30.0	12.6	12.3	70.9	1101
16	127.5	25.8	12.7	14.9	70.6	1500
17	60.0	30.0	19.1	13.0	60.2	2100
18	105.0	23.0	12.0	13.3	68.7	1700
19	60.0	23.0	17.4	13.0	59.0	2096
20	150.0	16.0	7.8	10.7	70.1	1042
21	91.5	30.0	16.3	12.3	59.6	2072

*Note:* Sample composition (70% cassava and 30% soy).

Abbreviations: MC, moisture content; PC, protein content; PD, protein digestibility.

The negative sign of the coefficient of BT (−5.03) indicates that the final moisture content of the product decreases when the extrusion temperature increases due to the fact that more water evaporates at higher temperatures. The positive sign of the coefficient of FMC (1.70) indicates that the moisture of the final product increases as the initial moisture content of the composite flour increases, and the positive sign of the interaction term (1.42) indicates that the combined increase in the two variables has a slightly synergistic effect on the moisture content of the final product. The above observations are in agreement with those observed in extruded cereal‐legume products by Guerrero et al. (2012) and Stojceska et al. (2009).

**FIGURE 1:**
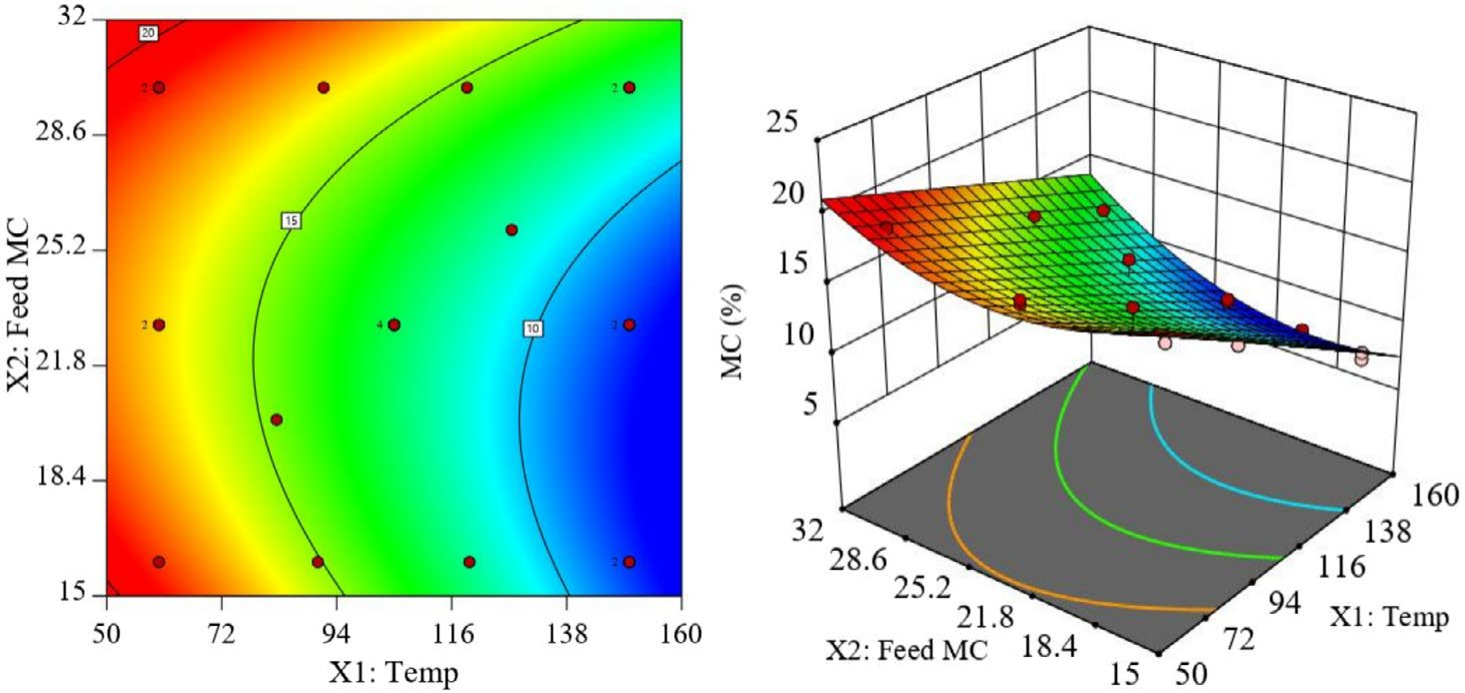
Contour (left) and response surface (right) plots of the model equation predicting variability of BT and FMC on extrudate moisture content.

Moisture contents of the extruded cassava‐soybean composite flours (7.8%–19.1%) were within the acceptable limits for complementary food products intended for storage, and according to international standards, products with moisture content below 15% are generally stable at ambient temperature because of restricted microbial growth, while products with 15%–20% moisture content are usually refrigerated to maintain safety and stability (Mosha and Bennink 2005). The contour plots (Figure 1) also suggest that an optimum moisture range of about 8%–12%, ideal for long shelf life without refrigeration, could be obtained at relatively high BTs and low feed moisture levels during extrusion.

#### 3.1.2 Protein Content

The protein content ranged from 10.6% ± 0.4% to 14.9% ± 0.6%. The highest protein content was found at the medium level (127.5°C, 25.8% moisture), while the lowest protein content was observed at the hottest and driest level (150°C, 16% moisture), and the model revealed the relationship between these levels and protein retention. The second‐order polynomial model (Equation 4) revealed the relationship between the levels and protein retention; therefore, the model explained 55% of the variation in protein content (*R* ^2^ = 0.545), thus the protein content was affected by the levels.

(4) $$\mathrm{PC}=13.59-0.46X_{1}+0.26X_{2}+0.56X_{1}X_{2}-1.40X_{1}2-0.75X_{2}2$$

Although the moderate value of *R* ^2^ indicates that the model only had moderate predictive power, all the terms in the model were significant (*p* < 0.05) and the lack‐of‐fit was not significant (*p* > 0.05), which means that the model can still be used to optimize the process.

**FIGURE 2:**
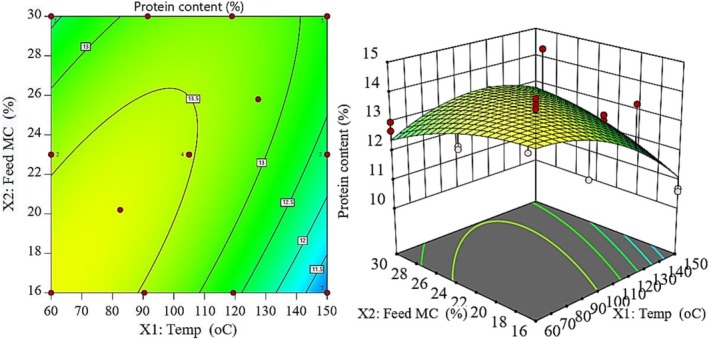
Contour (left) and response surface (right) plots of the model equation predicting variability of BT and FMC on protein content.

From Figure 2, the protein content significantly decreases as the BT approaches 150°C, especially at low feed moisture, because this is consistent with the negative linear coefficient for temperature (−0.46), which means that higher temperature results in more protein loss. The possible reasons for this include the Maillard reactions between reducing sugars and the ε‐amino group of lysine, heat‐induced denaturation and possible racemization of amino acids (Barrows et al. 2007). On the other hand, the positive coefficient for feed moisture (0.26) indicates that higher feed moisture level can help protect protein during extrusion, while higher moisture level can reduce shear stress and the effective temperature in the barrel. Moreover, the positive interaction term (0.56) suggests that high feed moisture level can partially counteract the detrimental effect of high BT, possibly due to the lower melt viscosity and mechanical shear with higher feed moisture level, which can reduce the local overheating and slow the Maillard reaction (Barrows et al. 2007; Oladiran and Emmambux 2022). Consequently, in this study, the Maillard reaction between cassava's reducing sugars and lysine in soybean appears to be the major reason for protein loss at high extrusion temperatures (Banaszkiewicz 2011; Sharma et al. 2014).

The results obtained in this study were consistent with the findings of Stojceska et al. (2009) and Guerrero et al. (2012) who observed a similar trend of protein loss in extruded legume‐based products, and in this study, all the formulations had protein content greater than or equal to the minimum protein content requirement for complementary foods recommended by WHO (13 g/100 g), except for samples processed under high temperatures and low moisture content. This underscored the need for proper control of extrusion conditions in order to minimize protein loss and maintain the nutritional quality of the extrudates.

#### 3.1.3 PD

The PD of the extruded cassava‐soybean samples varied between 58.3% ± 1.2% and 70.9% ± 0.9% (Table 3), and the highest PD was obtained at the combination of high BT and high feed moisture (150°C, 30% FMC), while the lowest PD was observed at low BT and low feed moisture (60°C, 16% FMC). The response surface model was found to be very predictive (*R* ^2^ = 0.864) with all model terms significant at *p* < 0.05 and lack of fit non‐significant at *p* > 0.05, indicating that the model adequately described the effect of the processing conditions on PD (Equation 5).

(5) $$\mathrm{PD}=67.70+6.93X_{1}+0.32X_{2}-0.35X_{1}X_{2}-2.06X_{1}2-3.92X_{2}2$$

**FIGURE 3:**
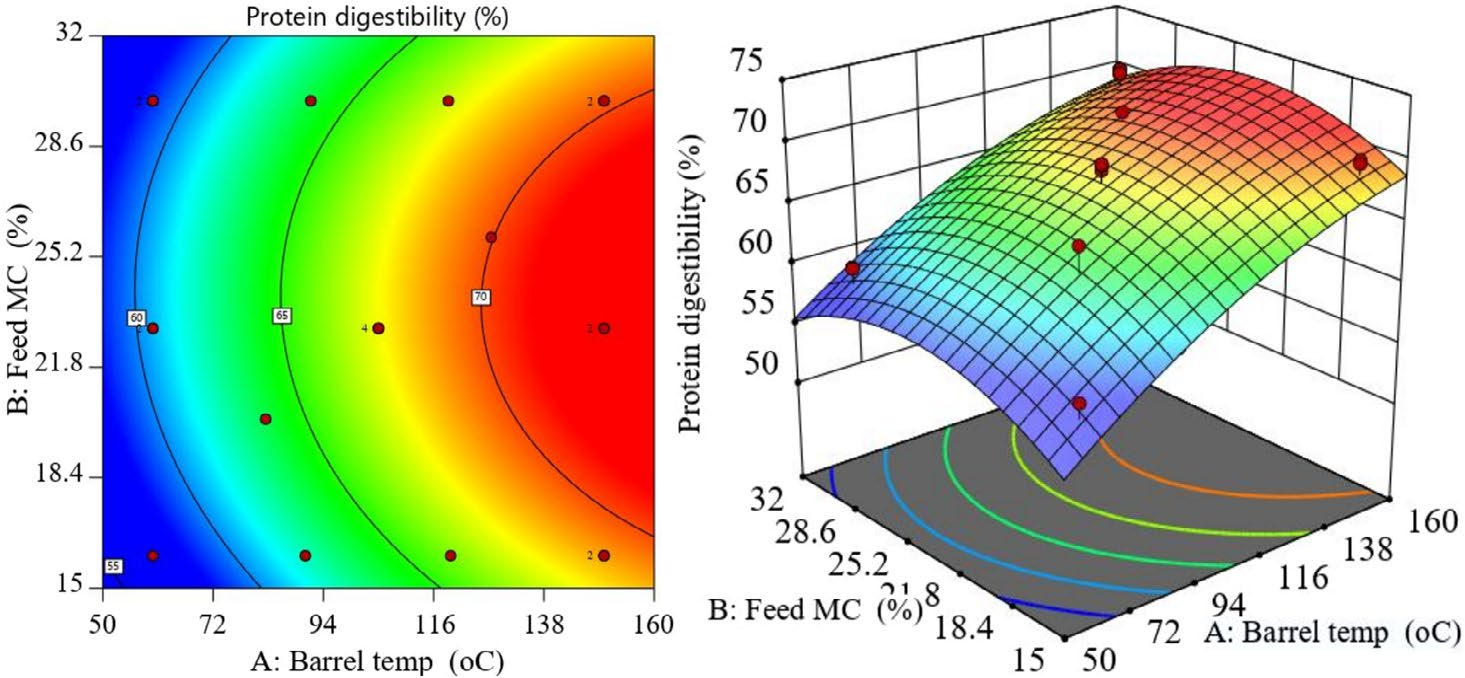
Contour (left) and response surface (right) plots of the model equation predicting variability of BT and FMC on product protein digestibility.

The contour and response surface plots in Figure 3 showed a positive relationship between BT and PD. PD increased progressively as BT rose from 60°C to 150°C. The large positive BT coefficient (6.93) indicates that higher extrusion temperatures strongly improved digestibility. This improvement is explained by protein unfolding, inactivation of soybean anti‐nutritional factors (including trypsin inhibitors and lectins), disruption of protein‐complex structures, and limited peptide hydrolysis under heat‐shear conditions (Abbas and Ahmad 2018; Licata et al. 2014).

The low but positive feed‐moisture coefficient (0.32) suggests that higher moisture had a small positive effect on PD, likely by supporting protein unfolding and reducing aggregation during extrusion. The negative quadratic terms indicate that digestibility declines when temperature or moisture moves beyond the optimum range. One likely reason is excessive protein aggregation. Another is increased formation of Maillard reaction products with lower digestibility. These findings agree with Licata et al. (2014), who reported improved digestibility in extruded cereal‐legume composites at higher temperatures.

#### 3.1.4 Viscosity

The viscosity of the extruded cassava–soybean porridges ranged between 1042 ± 28 and 2106 ± 45 cP, and the highest viscosity was found when extrusion was performed at a low BT and high feed moisture (60°C, 30% FMC), while the lowest viscosity was observed at a high BT and low feed moisture (150°C, 16% FMC). The fitted second‐order polynomial model (*R* ^2^ = 0.962; Equation 6) represented the relationship between the viscosity and processing conditions satisfactorily, indicating that the model is adequate to predict the effects of changes in temperature and moisture on porridge thickness.

(6) $$\begin{array}{c} \text{Viscosity}=1769.53-620.07X_{1}+94.98X_{2}+23.49X_{1}X_{2}\\ \;-370.12X_{1}2+129.16X_{2}2 \end{array}$$

The negative BT coefficient (−620.07) indicates that increasing extrusion temperature substantially reduced porridge viscosity. At higher temperatures, starch granules degraded and dextrinized. Cleavage of *α*‐1,4 and *α*‐1,6 glycosidic bonds generated shorter starch fragments with lower water‐binding capacity. As a result, the reconstituted porridge became thinner (Shevkani et al. 2017).

**FIGURE 4:**
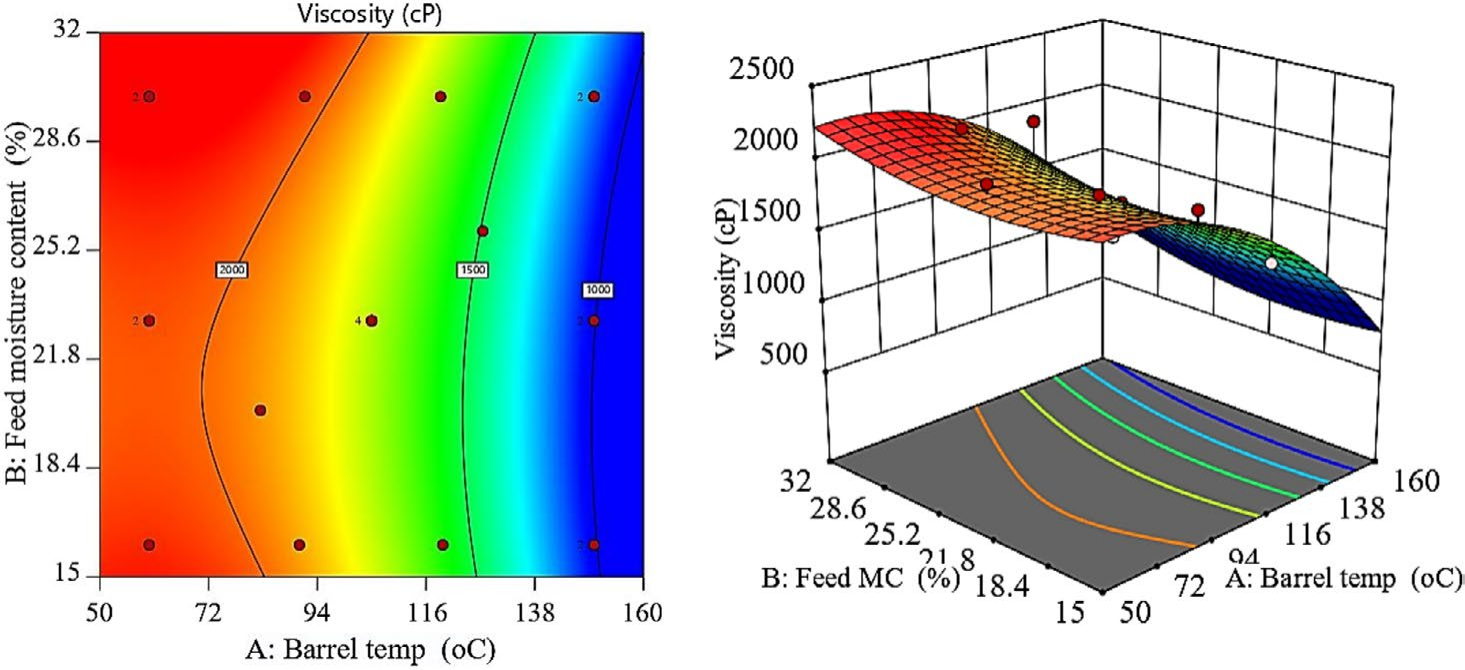
Contour (left) and response surface (right) plots of the model equation predicting variability of BT and FMC on product viscosity.

Conversely, the positive coefficient of feed moisture (94.98) suggests that higher feed moisture results in higher viscosity, because more water allows for starch gelatinization while preventing mechanical damage to starch, and larger, more intact starch structures remain, thus thickening the porridge. The interaction effect of temperature and moisture (23.49) indicates that both temperature and moisture have a modest effect on the viscosity of the porridge, and the effect of BT on the viscosity of the porridge is shown in Figure 4, where the viscosity decreased sharply with increasing BT. Contour plots are closely spaced along the temperature axis, indicating that BT has the highest effect on the reduction of viscosity, which is due to the mechanism of the extrusion process, where the starches are first gelatinized and then broken down into shorter chains under the effect of high temperature and shear. These shorter chains can bind less water and produce a thinner paste (Shevkani et al. 2017; Guerrero et al. 2012), and from a feeding perspective, viscosity is an important attribute of infant porridge, because high viscosity porridges are commonly diluted with excess water by caregivers, leading to a reduction in energy and nutrient density.

The World Health Organization recommends viscosity below 2500 cP for porridges given to children aged 6–12 months to ensure easy swallowing and choking risk, while maintaining good energy density (WHO 2009), and all porridges produced in this study were in the recommended range, while the best formulations had viscosities around 1000–1500 cP, ideal for infant feeding, and provided about 1 kcal/g of prepared porridge.

### 3.2 Optimization and Validation

The optimal extrusion conditions were determined by the desirability function with the BT at 145.6°C and the feed moisture at 19.7%, respectively, and the overall desirability value was 0.77. The protein content, PD, moisture content, and viscosity of the extrudate obtained under these conditions were 12.04%, 70.9%, 8.42%, and 1086.63 cP, respectively, because these results indicate a compromise among multiple requirements. Higher temperature and moisture increased the PD, but also increased the moisture content of the final product, which would lead to a shorter shelf‐life; thus, higher temperature and lower moisture maintained the dryness of the product and extended the shelf‐life, but resulted in excessive protein denaturation and loss.

Therefore, the desirability function provided an optimal solution, and it maintained the protein quality and digestibility with a low moisture content for storage and low viscosity for infants. Three confirmatory experiments were conducted at the optimum values (BT = 145.6°C, FMC = 19.7%), and the mean experimental values obtained were: protein content = 11.89% ± 0.32%, PD = 70.2% ± 1.1%, moisture content = 8.61% ± 0.28%, and viscosity = 1102.4 ± 34.7 cP. These values were within 5% of the predicted values (12.04%, 70.9%, 8.42%, and 1086.63 cP, respectively), with relative errors of 1.2%, 0.99%, 2.3%, and 1.5%, respectively (Table 4), confirming the validity of the model and its applicability under practical conditions.

**TABLE 4:** Optimization summary: predicted vs. experimental values for the optimized formulation (BT = 145.6°C, FMC = 19.7%; *n* = 3).

Response variable	Predicted value	Experimental value (mean ± SD, *n* = 3)	% error
Protein content (%)	12.04	11.89 ± 0.32	1.25
Protein digestibility (%)	70.9	70.2 ± 1.1	0.99
Moisture content (%)	8.42	8.61 ± 0.28	2.26
Viscosity (cP)	1086.63	1102.4 ± 34.7	1.45

#### 3.2.1 Sensory Evaluation Results

Evaluation of sensory qualities of the optimized cassava‐soybean porridge (BT = 145.6°C; FMC = 19.7%) was performed using a 9‐point hedonic scale (*n* = 30 panelists). Mean scores were 7.6 ± 0.8 for taste, 7.4 ± 0.9 for aroma, 7.5 ± 0.7 for color, 7.4 ± 0.7 for texture, and 7.3 ± 0.8 for overall acceptability. One‐way ANOVA indicated significant variation among tested products, and Tukey's HSD confirmed superior texture and overall acceptability for the optimized product (*p* < 0.05). The non‐optimized sample had the same 70:30 cassava‐soybean formulation but was processed at 120°C BT and 15% feed moisture content.

### 3.3 Nutritional and Practical Implications

#### 3.3.1 Nutritional Adequacy

The optimized cassava–soybean porridge offers clear nutritional advantages over traditional cassava‐only complementary foods. As shown in Table 5, age‐appropriate servings (25–50 g flour in 200 mL water) cover 12.4%–14.6% of daily energy and 34.1%–46.2% of daily protein requirements across the 6–8, 9–11, 12–23, and 24–59 month age groups, supporting its role as a complementary food alongside continued breastfeeding (WHO 2012). The in vitro PD of 70.9% indicates efficient protein utilization, enhanced by the lysine‐rich soybean complementing the characteristically low lysine content of cassava. Furthermore, the porridge viscosity of 1086.63 cP is well below the WHO‐recommended 2500 cP threshold, ensuring easy swallowability for young children while maintaining adequate energy density (306 kcal/100 g).

**TABLE 5:** Compliance of the optimized cassava‐soybean porridge with WHO complementary food guidelines by age group (*n* = 3 confirmatory batches).

Age group (months)	Serving size (g flour)	Energy per serving (kcal)	Protein per serving (g)	WHO energy requirement (kcal/day)	Energy coverage (% of daily)	Protein coverage (% of daily)
6–8	25	76.5	3.0	615	12.4	34.1
9–11	30	91.8	3.6	686	13.4	36.0
12–23	40	122.4	4.8	894	13.7	43.6
24–59	50	153.0	6.0	1046	14.6	46.2

*Note:* Energy and protein per serving calculated from optimized formulation (12.04% protein, 306 kcal/100 g). WHO energy requirements from WHO (2012). Protein requirements: 0.9 g/kg/day (WHO 2007). Servings assume 2–3 meals per day plus continued breastfeeding.

#### 3.3.2 Programmatic Implications

The porridge uses locally grown cassava and soybean, which supports local value chains while reducing dependence on imported complementary foods. The estimated production cost is UGX 3500 per 400 g pack (~USD 0.95), compared with UGX 8000–10,000 (~USD 2.16–2.70) for Cerelac. This corresponds to a consistent 56%–65% cost advantage, calculated from the price gap relative to the commercial comparator. At serving level (50 g flour in 200 mL water), the estimated cost is UGX 437 versus UGX 1000–1250. The laboratory‐scale estimate is based on 2024 Kampala prices, and further reductions are expected with scale‐up through improved process efficiency, bulk sourcing, and decentralized community‐level production.

#### 3.3.3 Process‐Structure–Function Relationships

Extrusion modified product functionality through linked protein and starch transformations. Heat‐shear‐moisture conditions promoted protein unfolding and reduced anti‐nutritional constraints, supporting improved digestibility. The same conditions disrupted starch crystallinity, fragmented amylose/amylopectin chains, and generated dextrins that lowered paste viscosity. These process‐structure changes explain the observed feeding‐relevant texture profile and improved nutrient delivery potential in the optimized porridge (Oladiran and Emmambux 2022; Masatcioglu et al. 2022).

The optimized ratio and processing conditions were established using NaroCas2 cassava and locally sourced Ugandan soybean cultivars. Because cassava starch characteristics and cyanogenic glucoside levels vary by variety, and soybean protein content differs across cultivars, re‐validation is recommended when raw materials change. Targeting narrower age bands would also require adjustment of formulation and portion guidance to align with age‐specific WHO requirements.

#### 3.3.4 Safety and Sensory Quality

Extrusion also contributed to product safety by reducing cyanogenic risk to acceptable levels under the optimized thermal‐shear conditions, consistent with Codex and East African safety limits. Sensory performance remained favorable, with strong acceptability and texture ratings from panelists. The non‐optimized sample had the same 70:30 cassava‐soybean formulation but was processed at 120°C BT and 15% feed moisture content. Compared with that non‐optimized comparator, the optimized product showed better texture acceptability while maintaining a low, age‐appropriate viscosity that supports practical infant feeding.

### 3.4 Limitations and Future Recommendations

The study has several limitations, because the sensory evaluation was done using semi‐trained university panelists and not the end users (caregivers/mothers of children aged 6–59 months), and so it may not be a true reflection of the acceptance of the porridge under normal feeding conditions. Second, residual HCN levels were not determined in the extruded product, and so we relied on the fact that the relatively high extrusion temperature (145.6°C) would degrade the cyanogenic glucosides to safe levels. Third, the amino acid profile and protein digestibility‐corrected amino acid score (PDCAAS) were not determined, which would have provided a better indication of protein quality, moreover, the bioavailability of micronutrients (e.g., iron and zinc) was not studied, despite the fact that phytates in soybean can affect mineral absorption. Fourth, there is no existing data from feeding trials to determine the true effect of the optimized porridge on the growth and nutritional status of the children, as well as its tolerability through digestion (such as flatulence, diarrhea, constipation). However, the in vitro analyses conducted revealed that the nutrient content and safety profile of the porridge are favorable, but further clinical feeding trials are required to confirm nutrient bioavailability and to ensure the absence of adverse gastrointestinal effects in the target population. Furthermore, the shelf‐life of the extruded product was not determined under tropical storage conditions, although the low moisture content (8.42%) suggests that the product will have a good storage life.

The study points out a number of lines for future research: Consumer acceptability testing using caregivers and young children in the target communities (as opposed to semi‐trained panelists), and measurement of residual HCN levels in the extruded flour and confirmed to be within WHO/FAO safety limits. The full amino acid profile and PDCAAS should be determined to evaluate protein quality, and micronutrient bioavailability (especially for iron and zinc) should be evaluated using in vitro digestion models, given the presence of phytates in soybean. Moreover, keeping quality of the product should be evaluated under conditions that mimic tropical storage (30°C–35°C and 75%–85% relative humidity), and feeding trials in children to demonstrate the effect of regular consumption of the porridge on growth and nutritional outcomes are needed. Additionally, testing simple fortification options (e.g., with iron, zinc or vitamin A) to enhance micronutrient intake should also be carried out, and there is a need to investigate how to scale up production and distribution in cassava growing regions of Uganda and East Africa in general, so that the product can be produced and sold locally, and also be affordable to households. Furthermore, community‐based acceptability studies among caregivers and mothers in target communities before scale‐up or programmatic distribution should be conducted, which would include examination of actual feeding practices, ease of preparation of the porridge, acceptance of the product by the child in the home, and caregivers' willingness and ability to pay, all of which are critical to adoption and long‐term sustainability.

## 4 Conclusion

In this research, an instant complementary porridge based on cassava and soybean was formulated for 6–59 month old children and the optimal formulation and extrusion window were determined. This product meets all WHO protein standards while being highly digestible and providing low moisture content that facilitates its storage stability and suitability for swallowing. Overall, this formulation proves the possibility of improving nutritional properties of cassava‐based complementary products by adding soybeans and proper extrusion technology.

In addition to technological improvements, this food product is also significant in that it has practical application value due to its high nutritional quality, sensory appeal, and relative inexpensiveness compared to popular commercial products. It could be implemented within existing public health programs, such as community kitchens, school feeding programs, and mother‐and‐child nutrition clinics, as a way to improve the nutritional situation among children. Further research is required, particularly concerning micronutrient absorption, storage life, and community acceptance.

## Author Contributions

**Catherine Byekwaso Arinaitwe:** conceptualization, investigation, methodology, writing – review and editing, writing – original draft, formal analysis. **Ivan Muzira Mukisa:** supervision, data curation, methodology, validation, visualization, formal analysis, software, investigation, conceptualization, writing – review and editing, writing – original draft. **Robert Mugabi:** conceptualization, investigation, methodology, writing – review and editing, writing – original draft, validation, formal analysis, software.

## Funding

The authors have nothing to report.

## Conflicts of Interest

The authors declare no conflicts of interest.

## Data Availability

The data that support the findings of this study are available from the corresponding author upon reasonable request.
